# Recent Advances in Interface Engineering for Planar Heterojunction Perovskite Solar Cells

**DOI:** 10.3390/molecules21070837

**Published:** 2016-06-25

**Authors:** Wei Yin, Lijia Pan, Tingbin Yang, Yongye Liang

**Affiliations:** 1 School of Electronic Science and Engineering, Collaborative Innovation Center of Advanced Microstructures, Nanjing University, Nanjing 210093, China; ericeyin@foxmail.com (W.Y.); ljpan@nju.edu.cn (L.P.); 2 Department of Materials Science and Engineering, South University of Science and Technology of China, Shenzhen 518055, China; liangyy@sustc.edu.cn

**Keywords:** perovskite solar cell, planar heterojunction, molecular interfacial materials

## Abstract

Organic-inorganic hybrid perovskite solar cells are considered as one of the most promising next-generation solar cells due to their advantages of low-cost precursors, high power conversion efficiency (PCE) and easy of processing. In the past few years, the PCEs have climbed from a few to over 20% for perovskite solar cells. Recent developments demonstrate that perovskite exhibits ambipolar semiconducting characteristics, which allows for the construction of planar heterojunction (PHJ) perovskite solar cells. PHJ perovskite solar cells can avoid the use of high-temperature sintered mesoporous metal oxides, enabling simple processing and the fabrication of flexible and tandem perovskite solar cells. In planar heterojunction materials, hole/electron transport layers are introduced between a perovskite film and the anode/cathode. The hole and electron transporting layers are expected to enhance exciton separation, charge transportation and collection. Further, the supporting layer for the perovskite film not only plays an important role in energy-level alignment, but also affects perovskite film morphology, which have a great effect on device performance. In addition, interfacial layers also affect device stability. In this review, recent progress in interfacial engineering for PHJ perovskite solar cells will be reviewed, especially with the molecular interfacial materials. The supporting interfacial layers for the optimization of perovskite films will be systematically reviewed. Finally, the challenges remaining in perovskite solar cells research will be discussed.

## 1. Introduction

Clean and renewable energy have become increasingly important for human society due to the increasing demand for energy and environmental concerns. Solar energy, which is abundant, widely distributed, and pollution-free, is one of the most important renewable energy sources. Solar cells represent a direct way to transform solar energy into electrical energy. Crystalline silicon-based solar cells are currently the dominant technology, with high power conversion efficiency (PCE) and stability, however, they suffer from relatively high production costs at large scale, resulting in only small-scale applications. Thin film solar cells based on copper-indium-gallium-selenide (CIGS) and CdTe could lower the cost, but have problems of material abundance. Organic solar cells, dye-sensitized solar cells (DSSC) and quantum dot solar cells display potential for low cost and easy of fabrication, but their performance is still not comparable to that of their traditional inorganic-counterparts.

**Figure 1:**
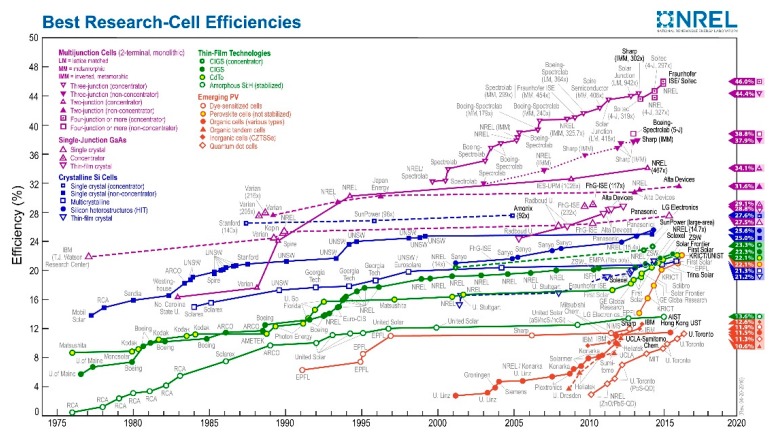
Solar cells efficiencies Reprinted with permission [14]. Copyright National Renewable Energy Laboratory.

**Figure 2:**
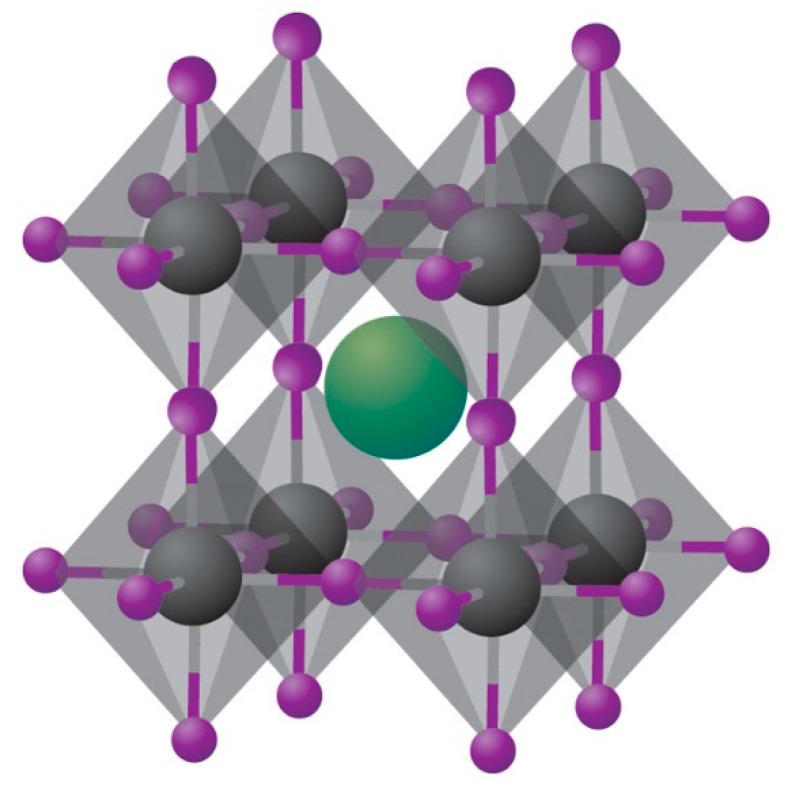
Crystal structure of cubic metal halide perovskites with the generic chemical formula ABX_3_. Organic or inorganic cations occupy position A (green) whereas metal cations and halides occupy the B (grey) and X (purple) positions, respectively. Reprinted from [10] with permission. Copyright 2014, rights managed by Nature Publishing Group.

Organic-inorganic hybrid perovskite solar cells are considered one of the most promising next generation photovoltaic technologies due to their high PCE, low cost and easy fabrication. Organic-inorganic hybrid perovskites have nearly all the good properties that a solar cell requires, including high absorption coefficients, low exciton binding energy, high charge-carrier mobility, long exciton diffusion length and easy tunable bandgap [1,2,3,4,5,6,7,8,9,10]. The PCEs of perovskite solar cells have rapidly increased from approximately 4% to over 20% in the past few years, which is by far the highest PCE among the novel solar cells [11,12,13]. The developments of different kinds of solar cells are depicted in Figure 1. Perovskite is the material described by ABX_3_, where X is an anion, and A, B are cations, respectively. Figure 2 shows the crystal structure of perovskite, where A = CH_3_NH_3_^+^, B = Pb^2+^ and X = Cl^−^, Br^−^, I^−^.

The original work for the development of perovskite solar cells was carried out in 2009 by Miyasaka et al., who first introduced CH_3_NH_3_PbI_3_ and CH_3_NH_3_PbBr_3_ as a photoactive layer, resulting in a PCE of 3.81% [11]. By optimizing the method of depositing the perovskite and electrolyte formulation, Park et al. increased the PCE to 6.5% [15]. Park and Grätzel replaced the liquid electrolyte with 2,2,7,7-tetrakis(*N,N*-di-*p*-methoxyphenylamine)-9,9-spirobifluorene (spiro-OMe TAD), and fabricated a sensitized all-solid-state perovskite solar cell, leading to an efficiency exceeding 9% [16]. Snaith et al. employed the mesoporous scaffold of Al_2_O_3_, further increasing the PCE to 10.9% by reducing energy losses. The mesoporous Al_2_O_3_ was reported to be an inert scaffold, which can force electrons to transport within the perovskite [7]. Etgar et al. proposed a hole conductor-free mesoscopic perovskite solar cell, which suggested that perovskite can act as a hole conductor in the perovskite solar cell [17].

The discovery of ambipolar properties for perovskite thin films allows for the development of planar heterojunction (PHJ) perovskite solar cells. A big breakthrough was carried out by Snaith et al., who fabricated a simple PHJ perovskite solar cell on a compact layer of TiO_2_ by a vapour-deposition method, resulting in a PCE of up to 15.4% [3]. By optimizing the method of depositing perovskite and using Y-doped TiO_2_ as the electron transporting layer, Yang et al. further improved the PCE to 19.3% [12]. By depositing a thin C_60_ or fullerene-derivative (acceptor) layer on the perovskite, Guo et al. reported an inverted PHJ perovskite solar cell [18]. With the employment of an inverted PHJ structure in perovskite solar cells, different perovskite casting methods and various contact materials are introduced to improve the PCE of perovskite solar cells [19,20,21]. A solution-based hot-casting method was reported to grow perovskites with millimeter-scale crystalline grains. As a result, the device with such large crystalline grains showed an efficiency approaching 18% in an inverted structure [22]. Recently, Huang et al. reported that by growing large-size perovskite grain on non-wetting hole transport layers, the PCE of inverted devices could be further improved to 18.3% [23].

**Figure 3:**
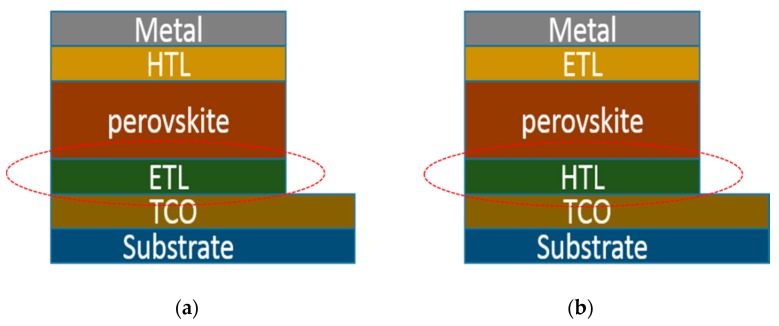
The device structure of conventional (**a**) and inverted (**b**) perovskite solar cell.

The use of conventional and inverted structures in PHJ perovskite solar cells eliminates the use of a mesoscopic metal oxide layer, which simplifies the fabrication process. The device structures of conventional and inverted PHJ perovskite solar cells are respectively depicted in Figure 3, where the transparent conductive oxide (TCO) and metal act as electrode, hole transport layer (HTL) and electron transport layer (ETL) function as interface modification. The perovskite itself takes on light harvest and charge transportation before the respective electrodes.

Although electrons and holes can transport within the perovskite, they are inclined to recombine with each other before reaching the respective electrodes. A number of methods have been employed to avoid the recombination of charges. Among them, interfacial engineering is one of the most valuable methods to depress charge recombination at the interface between the perovskite layer and the electrodes. On the one hand, the charge transporting properties and extraction can be engineered with the modification of energy-levels by appropriate interfacial layers. On the other hand, the film morphology of perovskite can be controlled by manipulating the supporting underlayer, leading to the improvement of film quality. Furthermore, the stability of perovskite solar cells can also be enhanced by the protection of interfacial layers [24].

Hence, interface engineering plays a critical role in the development of PHJ perovskite solar cells. A few reviews have been published that provide an overview on the rapid development of PHJ perovskite solar cells in the past few years, especially the efforts dedicated to optimize the perovskite film and the development of inorganic interfacial materials [25,26]. Only in the past few years, various interfacial materials, especially organic interfacial materials, have been developed to further improve the device performance and stability [27,28,29,30,31,32,33,34,35]. To systematically understand the role of interfacial layers in the fast development of perovskite solar cells, especially the novel molecular interfacial materials, which have the advantages of simple solution processing, a low-temperature annealing process, tunable work function, high mobility, and good compatibility with the perovskite film growth, we present an insightful overview of PHJ perovskite solar cells with interface engineering that was employed recently. Metal oxides, molecular interfacial materials, and other novel materials as interfacial layers will be discussed, respectively.

## 2. Metal Oxide

**Figure 4:**
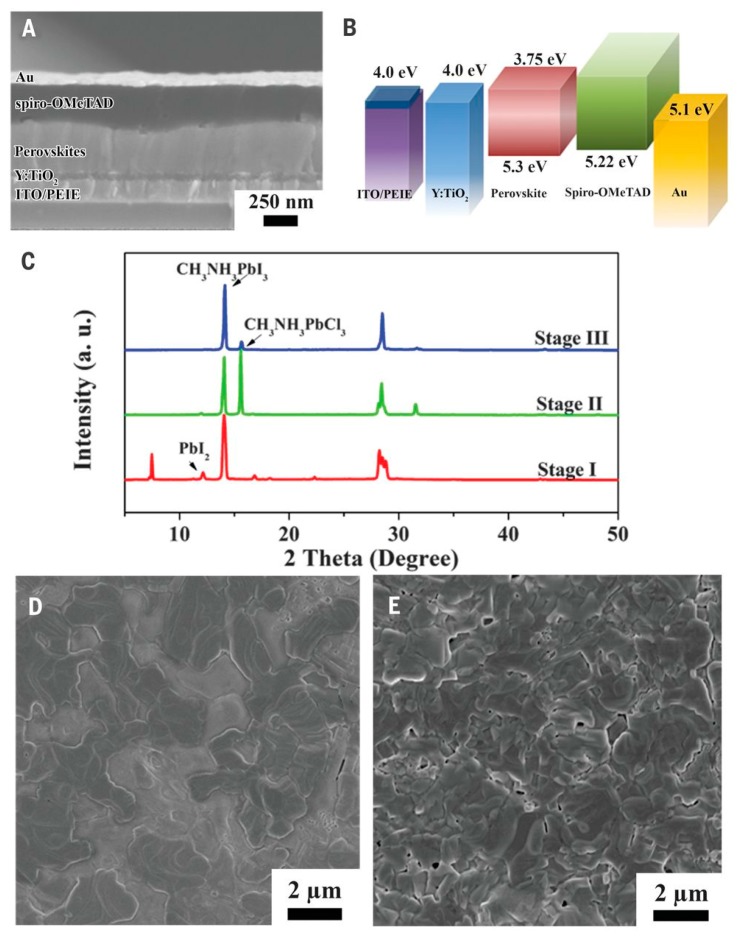
Structure and energy-level alignment of the perovskite solar cell. (**A**) SEM cross-sectional image of the device. The layers from the bottom are: (i) ITO/PEIE; (ii) Y-TiO_2_; (iii) perovskite; (iv) spiro-OMeTAD, and (v) Au; (**B**) Diagram of energy levels (relative to the vacuum level) of each functional layer in the device; (**C**) XRD patterns corresponding to perovskite film evolution with annealing time (stage I: 20 min, stage II: 60 min, stage III: 85 min); (**D**,**E**) Top-view SEM images of perovskite films at stage II (**D**) and stage III (**E**).

A conventional device structure for perovskite solar cell is TCO/ETL/perovskite/HTL/metal (Figure 3a). Generally, the TCO is fluorine tin oxide (FTO) or indium tin oxide (ITO). The ETL is generally a compact TiO_2_ layer, which works as a selective contact for electron collection at the anode [3,36,37]. The works used TiO_2_ based ETL are summarized in Table 1. Traditionally, a mildly acidic solution of titanium isopropoxide in ethanol is spin-coated on the FTO substrate followed by sintering at 500 °C, then a compact TiO_2_ is formed. Although the compact TiO_2_ shows good electron transporting properties, the processing is complicated and the high temperature required is not suitable for the fabrication of flexible solar cells. Therefore, low temperature and simple processed TiO_2_ are a requisite for low cost fabrication. Nanocrystalline rutile TiO_2_ was first introduced into perovskite solar cells by Yella et al., resulting in a high V_oc_ of 1.1 V [38]. The nanocrystalline rutile TiO_2_ was obtained at a low temperature of 70 °C using a hydrothermal method, in which the FTO substrate was immersed into a solution of TiCl_4_ and reacted at 70 °C. The device with nanocrystalline rutile TiO_2_ showed a PCE of 13.7%, significantly higher than that of the device with planar TiO_2_ obtained at 500 °C (PCE: 3.7%). The result was attributed to the nanocrystalline rutile TiO_2_, which can form an intimate junction with a large interfacial area. As a result, charge extraction was enhanced, leading to significant increase in device efficiency. To enhance electron selectivity, Yang et al. further modified the ETL for perovskite solar cells. On the one hand, they used ethoxylated poly-ethyleneimine (PEIE) to reduce the work function of ITO, leading to enhanced electron transport between the ETL and ITO layers (Figure 4). On the other hand, yttrium was doped into TiO_2_ (Y-TiO_2_), further enhancing electron transport and extraction. The Y-TiO_2_ ETL which was annealed at 150 °C, showed good conductivity of 2 × 10^−5^ S·cm^−1^, overcoming the low conductivity of the traditional TiO_2_. The Y-TiO_2_ ETL offers good Ohmic contact and balances charge transport, leading to a high PCE of 19.3% [12]. Different processes were also introduced to improve the properties of pristine TiO_x_, such as TiCl_4_ treatment, anodization, Zn-doping, y electron beam evaporation, and anodic oxidation, which resulted in a perovskite solar cell efficiency of around 15%. This is comparable with traditional TiO_2_-based devices, but is still lower than that of Y-TiO_2_–based devices [39,40,41,42,43,44].

The inferior electron mobility of pristine TiO_2_ is not an ideal electron transporting layer for highly efficient perovskite solar cells. Better candidates are expected for further enhancing the PCE of PHJ perovskite solar cells [45]. In contrast to TiO_2_, ZnO offers a higher electron mobility (200–300 cm^2^·V^−1^·s^−1^) at low temperature processing (requires no heating or sintering step), which makes it favorable for depositing on thermally sensitive substrates [46]. In addition, the ZnO solution-deposition process is simple, which makes it better choice over high-temperature deposited TiO_2_. PHJ perovskite solar cells using ZnO as an electron transporting layer exhibited a PCE as high as 15.7%. Furthermore, flexible devices that incorporated ZnO, also showed good performance, with a PCE of 10% [47].

**Figure 5:**
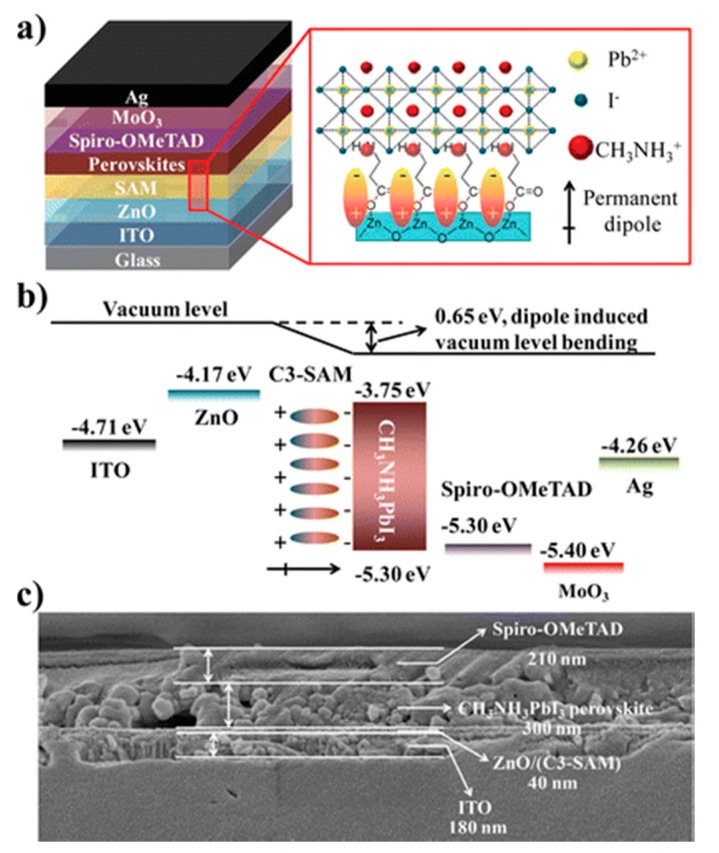
(**a**) Schematic diagram of a perovskite solar cell device structure, SAM induced permanent dipole formation, and involvement of the SAM in the crystalline structure of perovskite crystals; (**b**) Schematic energy level of each layer in perovskite solar cell; (**c**) Cross-section SEM image of the PSC device (without MoO_3_/Ag). Reprinted from [48] with permission. Copyright 2015, American Chemical Society.

The morphology of perovskite film is not only determined by its internal growth mechanism, but is also related to the surface properties of the substrates. A method for modifying ZnO-coated substrates was reported using a self-assemble monolayer (SAM) C3-SAM [48] (Figure 5). The C3-SAM on the sol-gel ZnO layers would induce significant improvements in the morphology of the perovskite film due to the enhanced wetting of perovskite on the ZnO, where the amino group was expected to change into ammonium by hydrogen ion exchange and promotes the crystalline structure of perovskite. Additionally, the C3-SAM also can tune the work function of the ZnO surface, which would improve the energy-level alignment and enhance electronic coupling between the ZnO and perovskite. As a result, charge transportation and extraction can be improved. With the introduction of the C3-SAM, highly crystalline perovskite films were formed, and better energy-level alignment was available, significantly increasing the device efficiency from 11.96% to 15.67%. A fullerene derivative (PC_70_BM) was also introduced to modify the surface of ZnO as ETL, since charge transfer from perovskite to PC_70_BM is very fast. The device used ZnO/PC_70_BM as electron transport layer allowing the efficient collection and dissociation of a larger number of excitons [49].

Indium oxide (In_2_O_3_), as a promising n-type semiconductor material, has been widely employed in optoelectronic applications. A low-temperature solution-processed In_2_O_3_ nanocrystalline film was introduced as an ETL in PHJ perovskite solar cells [50]. By taking the advantages of high mobility, wide band gap, and high transmittance of In_2_O_3_, the PHJ perovskite solar cells using In_2_O_3_ as an ETL achieved an efficiency exceeding 13%.

In addition, to further modify the surface of In_2_O_3_ film with PCBM molecule, the pinholes or cracks along In_2_O_3_ grain boundaries were deactivated, further reducing the charge recombination. As a result, the efficiency of In_2_O_3_-based PHJ perovskite solar cells was improved to 14.83%, with J_sc_ of 20.06 mA·cm^−2^, V_oc_ of 1.08 V, and FF of 0.685. Other metal oxides, such as SnO_2_, were also introduced in PHJ perovskite solar cells as an ETL. The corresponding devices showed an promising efficiency of 16.92% [51].

Another device structure for PHJ perovskite solar cells is TCO/HTL/perovskite/ETL/metal (Figure 3b). The HTL generally is poly(3,4-ethylenedioxythiophene):poly(styrenesulfonate) (PEDOT:PSS), which is hygroscopic and acidic, making it unsuitable for stable perovskite solar cells. In addition to functioning as a cathode interface, metal oxides can also be used as anode interfaces in PHJ perovskite solar cells. To avoid the use of acidic interfacial layers, NiO_X_ was introduced as an anode buffer layer to replace PEDOT:PSS [52,53]. Although better energy-level alignment was observed using NiO_X_, the perovskite cannot form a continuous film on the NiO_X_. As a result, the ETL (e.g., PCBM) may contact directly with NiO_X_ through the pinholes. To improve the surface properties, the NiO_X_ was treated with UV-ozone, whereby the resulting device showed a PCE of 7.8%. However, the performance of perovskite solar cells based on NiO_X_ HTL is still not satisfactory when compared with other HTLs due to the low FF and J_sc_ [29,54]. To solve this problem, doping was employed to improve the conductivity of NiO_x_ [55]. Copper was used to dope in NiO_X_ (Cu-NiO_X_) with a solution processing method, which is simple and allows for the fabrication of high performance perovskite solar cells due to the improved electrical conductivity and favorable perovskite crystallization [28]. The devices based on Cu-NiO_X_ showed a high PCE of 15.4%, with J_sc_ of 19.01 mA·cm^−2^, V_oc_ of 1.11 V, and FF of 0.73. This result indicated that less potential losses can be achieved with Cu-NiOx as an HTL, which confirms the promising applicability of Cu-NiO_X_ in perovskite solar cells. However, the Cu-NiO_X_ HTL has to be annealed at temperatures above 400 °C in order to achieve high crystallinity. Recently, a low-temperature, Cu-NiO_X_ hole-transporting layer was reported [56]. The resulting perovskite solar cells exhibited a high PCE up to 17.8%, with J_sc_ of 22.23 mA·cm^−2^, V_oc_ of 1.05 V, and FF of 0.76. A low-temperature processed NiO-based nanocrystal ink (LT-NiO) was introduced as an HTL, which increased the efficiency to 17.5%, with J_sc_ of 20.57 mA·cm^−2^, V_oc_ of 1.111 V, and FF of 0.77 [57]. Additionally, a solution-derived NiOx or NiOx nanoparticle was also introduced as an HTL to replace PEDOT:PSS, the devices showed the efficiency of 16.47% and 16.1%, respectively, and the device performance were retained for up to 60 days [58,59].

Besides NiO_X_, another p-type metal oxide, VO_X_, which shows high transmittance and quenching efficiency with perovskite, was introduced as an HTL in inverted PHJ perovskite solar cells [63]. Firstly, the numerical value of x in VO_X_ was 2.428, indicating the good conductivity due to the presence of oxygen vacancy. Secondly, the VO_X_ layer was able to enhance the wettability of the substrate surface. Thirdly, the valence band energy level of VOx is 5.36 eV, which can be matched well with perovskite film. Fourthly, the steady-state photoluminescence (PL) of perovskite thin films on the VO_X_ indicated that holes could be effectively extracted from perovskite. Finally, with a solvent-assisted process, perovskite thin film can be formed with high percentage coverage. In view of the integrated properties of the VO_X_, the corresponding inverted PHJ perovskite solar cells showed a PCE of 14.24%, with J_sc_ of 22.29 mA·cm^−2^, V_oc_ of 0.9 V, and FF of 0.71. Other p-type metal oxide, MoO_X_, was also introduced in inverted PHJ perovskite solar cells as an HTL, which showed a PCE of 6.5% with a J_sc_ of 16.5 mA·cm^−2^, V_oc_ of 0.96 V, and FF of 0.41, much lower than the efficiencies mentioned above [62].

## 3. Molecular Interfacial Materials

Molecular interfacial materials were introduced in PHJ perovskite solar cells only in the past few years, offering a promising method to improve the performance. The details are summarized in Table 2. PEDOT:PSS is usually used in organic electronics because of its simple solution processing, planarization effect on the underlying ITO layer, and a low-temperature annealing process [64]. In polymer solar cells, the PEDOT:PSS has been widely used as an HTL [65,66]. The PEDOT:PSS was first introduced in perovskite solar cells by Guo et al., who employed a glass/ITO/PDOET:PSS substrate as the anode, CH_3_NH_3_PbI_3_/fullerene(C_60_) as an active layer, and BCP/Al as the cathode. The corresponding device showed an efficiency of 3.9% [18]. Soon after, various perovskite casting methods were employed to improve the PCE to about 18%. To study the role of HTL (e.g., PEDOT:PSS), HTL-free inverted perovskite solar cells were fabricated. The devices achieved a remarkable efficiency of 12.5%, with J_sc_ of 16.1 mA·cm^−2^, V_oc_ of 0.99 V, and FF of 0.739. This is promising though it is still not satisfactory when compared with traditional PEDOT:PSS HTL due to the low FF and J_sc_ [67,68]. This indicated that HTL is critically important in efficient perovskite solar cells. Various additives, such as grafted sulfonated-acetone-formaldehyde lignin (GSL), TiO_2_/MoO_3_, and nanoparticle MoO_X_ were introduced to dope PEDOT:PSS, which are expected to increase the conductivity of HTL and modify the morphology of perovskite films. The corresponding devices showed efficiencies of 14.94%, 15.79%, and 13.63%, respectively [69,70,71].

**Figure 6:**
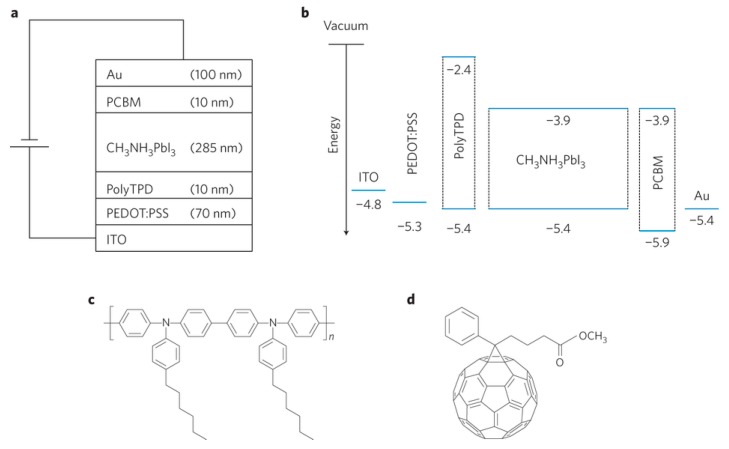
(**a**) Stacked layer structure; (**b**) Schematic of the relative energy levels of each layer; (**c**) Chemical structure of the polyarylamine (polyTPD); (**d**) Chemical structure of PCBM. Reprinted from [30] with permission . Copyright 2013, rights managed by Nature Publishing Group.

Although the perovskite can form good film quality on the ITO/PEDOT:PSS, the work function of PEDOT:PSS cannot match well with the perovskite. In addition, the ambipolar properties of perovskite itself may allow the electrons from the perovskite to inject into the ITO/PEDOT:PSS electrode [75]. PolyTPD were selected for electron-blocking layers as its appropriate highest occupied molecular orbital (HOMO) and lowest unoccupied molecular orbital (LUMO) levels, can match well with the valence band and conduction band of the perovskite, respectively, allowing for efficient hole transport from perovskite to polyTPD (Figure 6). As the LUMO of polyTPD is closer to vacuum level than the conduction band of perovskite, polyTPD can efficiently block the flow of electrons from perovskite. The corresponding device with polyTPD showed an efficiency of 12.04% and a V_oc_ of 1.05V [30]. Additionally, since the work function of PEDOT:PSS is lower than the ionization potential of perovskite, the potential energy loss would present at the PEDOT:PSS/perovskite interface. By the introduction of polyTPD, the energy loss in perovskite solar cells could be decreased. To modify the interface between the perovskite and the ITO electrode, a self-organized HTL (SOHTL), which is composed of PEDOT:PSS and perfluorinaated (PFI), was introduced in perovskite solar cells. This SOHTL affords a good energy level alignment with the ionization potential of perovskite, which could reduce the potential energy loss at the PEDOT:PSS/perovskite interface [73]. A polymeric material obtained from the copolymerization of 1,4-bis(4-sulfonatobutoxy)benzene and thiophene moieties (PhNa-1T) was also deposited on PEDOT:PSS. The incorporation of PhNa-1T into to the HTL, charge extraction from the perovskite to HTL was enhanced and charge recombination in the bulk perovskite and HTL/perovskite interface were thus suppressed. As a result, the flexible perovskite solar cells achieved a high efficiency of 14.7%, with Jsc of 18.4 mA·cm^−2^, V_oc_ of 1.03 V, and FF of 0.774. More importantly, the PhNa-1T interlayer allowed perovskite solar cells to have better stability than PEDOT:PSS in air [74].

**Figure 7:**
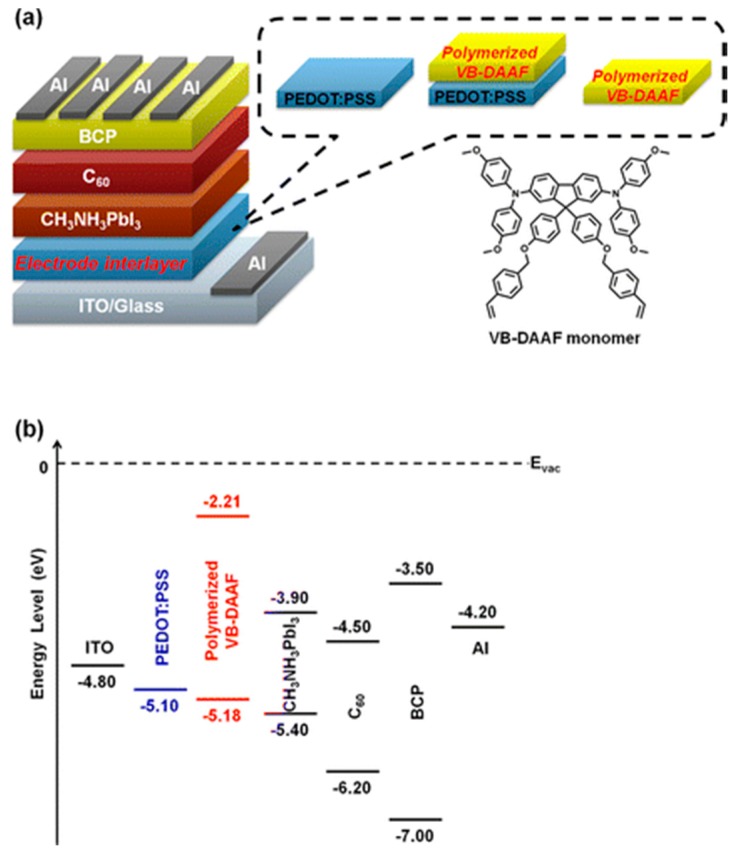
(**a**) Device configuration of the hybrid solar cell in this study of glass/ITO/electrode interlayer/CH_3_NH_3_PbI_3_ perovskite/C_60_/BCP/Al. The inset depicts the molecular structure of the VB-DAAF **monomer**; (**b**) Diagrams the energy levels of each layer. Reprinted with permission [24]. Copyright 2015, American Chemical Society.

Recently, a functional p-type, polymerized organic electrode interlayer was reported [24] (Figure 7). The styrene-functionalized 9,9-diarylfluorene-based triaryldiamine monomer (VB-DAAF) was directly cast on the substrates by a spin-coating process. Then, the uniform and flat polymerized VB-DAAF HTL was formed after thermal annealing at 195 °C.

**Figure 8:**
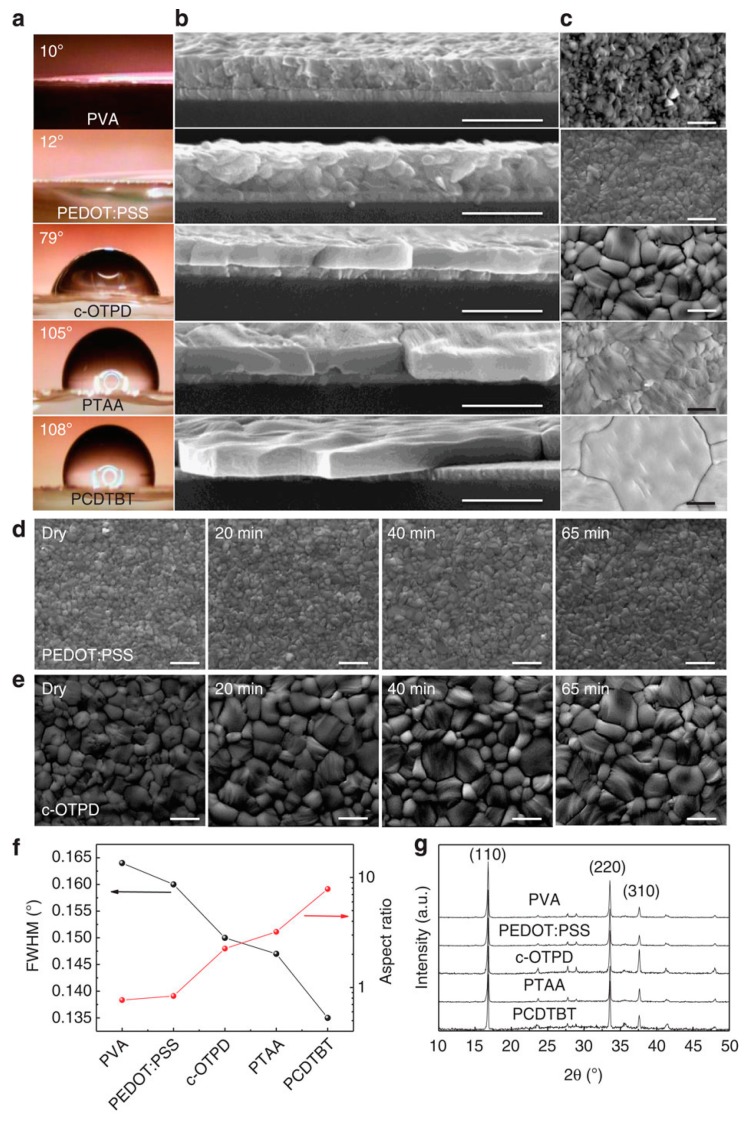
The contact angle of water on the varied HTLs (**a**), the cross-section SEM (**b**), top-view SEM (**c**) and X-ray diffraction patterns of the 360-nm MAPbI_3_ grown on PVA-, PEDOT:PSS-, c-OTPD-, PTAA- and PCDTBT-covered ITO substrates (**g**). Scale bars, 1 μm in b,c; (**d**,**e**) the top-view SEM images of the MAPbI_3_ grown on PEDOT:PSS (top row) and c-OTPD (bottom row) right after drying and after 20, 40 and 65 min of thermal annealing at 105 °C. Scale bar, 1 μm; (**f**) HTL-dependent X-ray diffraction (110) peak full width at half maximum (FWHM) and average grain size/thickness aspect ratio of the MAPbI_3_. Reprinted with permission. Copyright 2015 macmillan publishers limited.

The polymerized VB-DAAF HTL exhibited good energy-level alignment with the valence-band-edge level of perovskite, enhancing hole transportation. The large energy barrier between the polymer and perovskite in the conduction-band-edge level effectively blocked electrons from reaching the positive electrode and reduced the photon energy loss due to recombination. By depositing VB-DAAF on PEDOT:PSS, the recombination of photo-generated charge carriers was reduced and the energy loss was decreased, resulting in the V_oc_ increasing from 0.85 V to 0.99 V and the J_sc_ increasing from 16.37 mA·cm^−2^ to 21.53 mA·cm^−2^. Meanwhile, the removal of PEDOT:PSS decreased the series resistance of the device and further enhanced the efficiency of solar cells to 15.17%. To overcome the acid PEDOT:PSS, a pH-neutral and low-temperature deposited conjugated polyelectrolyte poly[2,6-(4,4-bis-potassium butanylsulfonate-4*H*-cyclopenta-[2,1-b;3,4-b’]-di-thiophene)-*alt*-4,7-(2,1,3-benzothiadiazole)] (CPE-K) was also introduced to fabricate PHJ perovskite solar cells, resulting in a PCE of 12.51% [60,76]. Moreover, poly(3-methylthiophene) (PMT), poly(thiophene) (PT), poly(3-bromothiophene) (PBT) and poly(3-chlorothiophene) (PCT) were also introduced to replace PEDOT:PSS, resulting in PCEs of 12.3%, 15.8%, 16.3%, and 16.5%, respectively [61]. Previous results indicated that it is difficult for perovskite to form a continuous film on the non-wetting substrate. Nevertheless, this issue was overcome by Huang et al., who developed a new method to grow perovskite film with large crystalline grains on non-wetting HTL [23]. The resulting large crystalline grains in perovskite reduced the grain boundaries which could cause charge recombination due to the presence of large density of charge traps [12,77,78,79,80]. The perovskite device with large-size grain showed a PCE of 18.3% due to enhanced J_sc_, V_oc_ and FF (Figure 8). The reason was attributed to the surface tension dragging force from the wetting PEDOT:PSS substrates, which could reduce the grain boundary mobility. However, the dragging force can diminish if the substrate is non-wetting, which enables the growth of larger grains, yielding higher grain boundary mobility. They found that non-wetting hole transporting layers can increase nucleus spacing by suppressing heterogeneous nucleation and facilitate grain boundary migration in grain growth by imposing less drag force. Thus, non-wetting hole transporting layers are favorable for the growth of perovskite grain with high average aspect ratio. Consequently, all photo-generated charges could diffuse to the charge transport layers over grain boundaries without recombination, and the device efficiency was only determined by the charge recombination at grain boundaries or at electrode interfaces. Also studied different non-wetting substrates with several polymers. When a crosslinked *N*4,*N*4′-bis(4-(6-((3-ethyloxetan-3-yl)methoxy)hexyl)phenyl)-*N*4,*N*4′-diphenylbiphenyl-4,4′-diamine(c-OTPD) or poly(bis(4-phenyl)(2,4,6-trimethylphenyl)amine) (PTAA) was used as a HTL, the PHJ perovskite solar cells showed efficiencies of 17.8% and 18.1%, respectively. The PTAA allowed for the presence of larger perovskite grains and had higher work function than that with c-OTPD. By doping a strong electron acceptor 2,3,5,6-tetrafluoro-7,7,8,8-tetracyanoquinodimethane (F4-TCNQ) in PTAA for enhancing the conductivity, the device with non-wetting PTAA HTL showed a high PCE of 18.3%. These works demonstrated that the use of non-wetting HTLs is an effective way to improve the efficiency of the devices. In addition, the non-essential of acidic PEDOT:PSS is expected to achieve better stability.

## 4. Other Interface Materials

Besides the interface materials mentioned above, other emerging novel interface materials are also developed for PHJ perovskite solar cells, including graphene oxide and inorganic materials [81,82,83]. These materials have shown promising characteristics in device efficiency and stability (Table 3).

**Figure 9:**
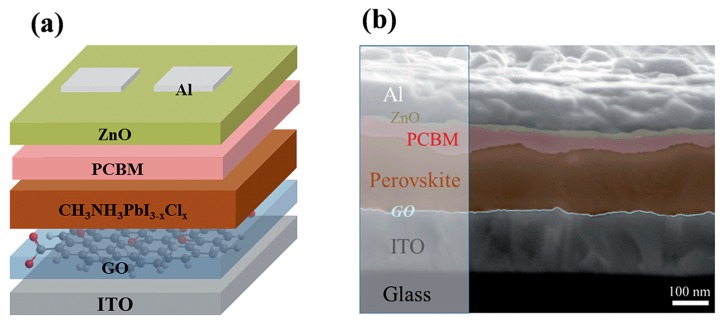
(**a**) Schematic of the inverted photovoltaic device configuration consisting of a structure of ITO/GO/CH_3_NH_3_PbI_3−_**xClx**/PCBM/ZnO/Al; (**b**) Cross-sectional SEM image of the optimized inverted device configuration. Reprinted with permission [82]. Copyright 2014, Royal Society of Chemistry.

Graphene oxide was introduced to be a hole transporting layer in polymer solar cells for enhancing efficiency and stability because of its suitable work function and acceptable vertical resistivity or its surface effect on the active layer [84,85,86]. Graphene oxide, an intimate graphene derivative, was also introduced into PHJ perovskite solar cells as a hole conductor by replacing the acidic PEDOT:PSS (Figure 9). The graphene oxide layer can efficiently extract hole from perovskite, and facilitate the formation of homogenous large domains as well as surface coverage. By optimizing thickness of the graphene oxide layer, balanced charge transport within the perovskite was achieved, which contributed to the improvement of $J_{\text{sc}}\;$ and FF.

Furthermore, the presence of graphene oxide allows the perovskite film to grow into larger textured domains, resulting in a complete coverage. The device with graphene oxide as a HTL, showed a high PCE of 12.5%, which is comparable to the cells using the conventional PEDOT:PSS. The GO/PEDOT:PSS hybrid bilayer HTL was successfully developed for inverted PHJ perovskite solar cells, where GO layer can efficiently extract holes out of perovskite and block electrons at ITO/PEDOT:PSS interlayer from recombination, leading to an efficiency of 13.1% [87]. Furthermore, an ammonia modified graphene oxide (GO:NH_3_) was introduced into PEDOT:PSS. The resulting PEDOT:PSS-GO: NH_3_ HTL-based inverted PHJ perovskite solar cells achieved a high PCE of 16.11% [61]. A hysteresis-free planar perovskite solar cell with a PCE of 19.1% was achieved by using a room-temperature vacuum-processed C_60_ ETL [74].

High efficiency of perovskite solar cells generally can be achieved at a small effective device area (e.g., <0.1 cm^2^), but poor stability is often observed. Although a number of studies have reported the fabrication of centimeter-scale perovskite solar cells, the efficiency obtained from those devices is inferior [88,89]. NiMgLiO was introduced as a HTL to replace PEDOT:PSS due to its high conductivity of 2.32 ×10^−3^ S cm^−1^. The NiMgLiO based HTL offered Ohmic contact at the FTO-perovskite interface by decreasing the barrier height through the staircase energy level alignment, and enhanced hole extraction was obtained. With the NiMgLiO based HTL, a large-size (1.02 ${\text{cm}}^{2}$) perovskite solar cell with an efficiency of up to 16.2% was achieved. Furthermore, hyteresis in the current-voltage characteristics was eliminated, with 90% of the initial PCE remaining after 1000 hours light soaking. Other inorganic materials, CdS and CuS, were introduced as ETL, HTL respectively in conventional and inverted PHJ perovskite solar cells, which achieved PCEs of 12.2% and 16.2%, respectively [90,91].

## 5. Summary and Outlook

The unique combination of virtually all the good properties required in a solar cell provides perovskite solar cells with excellent performance over other thin-film solar cells. This review has highlighted interface engineering of the layer under the perovskite film in different materials, especially the role of different molecules on perovskite solar cells. Efforts dedicated towards optimizing the interface included the following four aspects: (1) better alignment of the interfacial work function with perovskite, which can improve the transfer of charges and increase the device V_oc_; (2) high charge extraction and transport capacity; (3) interfacial properties to optimize the perovskite film growth; and (4) new ETL/HTL materials to improve the device stability. Molecular interfacial materials, which have the advantages of simple solution processing, low-temperature annealing process, and tunable electrical as well as optical properties, offer a bright future for the optimization of PHJ perovskite solar cells.

Though device efficiency and stability have been significantly improved, many issues still remain to be solved before perovskite solar cells can be used in real applications. The challenges are contained as the following aspects: (1) film morphology control of perovskite. In spite of efforts dedicated to optimize the perovskite film such as solution-based hot-casting [22], vapor-deposition [3], additive optimization [19,92,93], solvent optimization [94,95], solvent annealing [20], and perovskite precursor solution optimization [96,97] to control the high quality perovskite thin film growth, the complex procedures of perovskite film preparation make the availability of uniform and large size perovskite films difficult. Meanwhile, there are still many problems with no reasonable explanation in perovskite solar cells such as hysteresis and S-shaped current-voltage characteristics; (2) device stability. The stability of solar cells is the key to realize real applications. In spite of efforts dedicated to develop novel interfacial materials, the stability of perovskite solar cells is far inferior to that of traditional crystalline silicon solar cells. The molecular interfacial materials, which show good compatibility with perovskite, provide a promising way to further improve the stability of perovskite solar cells; (3) Pb. The presence of Pb in perovskite solar cells restricts the further large-scale applications due to the potential environmental pollution and health damage. Sn^2+^, Cu^2+^ and Fe^2+^ are the potential candidates to replace Pb. However, due to the limit of low mobility and diffusion length, as well as the poor stabilisation of lead-free perovskite material, the efficiency of lead-free perovskite solar cells is still obviously lower than traditional lead-based perovskite solar cells [98,99,100].

With rapid development of perovskite solar cells, commercial applications in the near future are expected by sequentially solving the challenges mentioned above, opening up a new way for efficient, low-cost and flexible power generation.

## Figures and Tables

**Table 1:** Device characteristics of representative PHJ perovskite solar cells employing metal oxide as the supporting layer.

Device Structure	V_oc_ (V)	J_sc_ (mAcm^−2^)	FF	PCE (%)	Ref.
ITO/TiCl-TiO_2_/CH_3_NH_3_PbI_3−x_Cl_x_/Spiro-OMeTAD/Au	1.09	19.7	0.759	16.4	[39]
FTO/A-TiO_2_/CH_3_NH_3_PbI_3_/Spiro-OMeTAD/Ag	1.06	20.5	0.7	15.2	[40]
FTO/Zn-TiO_2_/CH_3_NH_3_PbI_3_/Spiro-OMeTAD/Ag	1.04	23.83	0.649	16.07	[41]
ITO/AO-TiO_2_/CH_3_NH_3_PbI_3−x_Cl_x_/Spiro-OMeTAD/Au	1.00	19.08	0.71	13.47	[44]
FTO/PW_12_-TiO_2_/CH_3_NH_3_PbI_3−x_Cl_x_/Spiro-OMeTAD/Au	1.1	20	0.7	15.45	[43]
ITO/E-TiO_X_/CH_3_NH_3_PbI_3_/P3HT/MoO_3_/Ag	0.93	27.8	0.57	14.7	[42]
FTO/Zn-TiO_2_/CH_3_NH_3_PbI_3_/Spiro-OMeTAD/Au	1.05	19.8	0.64	13.7	[38]
ITO/PEIE/Y-TiO_2_/CH_3_NH_3_PbI_3−x_Cl_x_/Spiro-OMeTAD/Au	1.13	22.75	0.75	19.3	[12]
ITO/ZnO/ CH_3_NH_3_PbI_3_/Spiro-OMeTAD/Ag	1.03	20.4	0.75	15.7	[47]
ITO/ZnO/SAM/ CH_3_NH_3_PbI_3_/Spiro-OMeTAD/Ag	1.07	22.5	0.65	15.67	[48]
ITO/In_2_O_3_/CH_3_NH_3_PbI_3_/Spiro-OMeTAD/Au	1.07	17.9	0.68	13	[50]
ITO/In_2_O_3_/PCBM/ CH_3_NH_3_PbI_3_/Spiro-OMeTAD/Au	1.08	20.06	0.685	14.83	[50]
ITO/SnO_2_/(FA_0.85_MA_0.15_Pb(I_0.85_Br_0.15_)_3_/Spiro-OMeTAD/Au	1.09	23.06	0.68	16.92	[51]
ITO/Cu-NiO_X_/CH_3_NH_3_PbI_3_/C_60_/Bis-C_60_/Ag	1.12	19.16	0.73	15.4	[60]
ITO/Cu-NiO_X_/CH_3_NH_3_PbI_3_/C_60_/Bis-C_60_/Ag	1.05	21.6	0.77	17.46	[56]
ITO/NiO_X_/CH_3_NH_3_PbI_3_/ZnO/Al	1.01	21	0.76	16.1	[59]
ITO/LT-NiO/CH_3_NH_3_PbI_3−x_Cl_x_/PCBM/PDINO/Ag	1.111	20.57	0.77	17.5	[57]
ITO/NiO_X_/CH_3_NH_3_PbI_3_/PCBM/Ag	1.07	20.58	0.748	16.47	[61]
ITO/MoO_X_/CH_3_NH_3_PbI_3_/PCBM/C60/BCP/Al	0.96	16.5	0.41	6.5	[62]
ITO/VO_X_/CH_3_NH_3_PbI_3_/PCBM/C60/BCP/Al	0.9	22.29	0.71	14.23	[63]

**Table 2:** Device characteristics of representative PHJ perovskite solar cells employing molecular interfacial materials as the supporting layer.

Device Structure	V_oc_ (V)	J_sc_ (mAcm^−2^)	FF	PCE (%)	Ref.
FTO/PEDOT:PSS/CH_3_NH_3_PbI_3−x_Cl_x_/PCBM/Ag	0.94	22.4	0.83	17.47	[22]
ITO/PEDOT:GSL/CH_3_NH_3_PbI_3_/PCBM/Al	1.03	20.1	0.72	14.94	[69]
ITO/TiO_2_-MoO_3_-PEDOT:PSS/CH_3_NH_3_PbI_3−x_Cl_x_/C_60_/Bphen/Ag	0.96	17.35	0.84	13.63	[71]
ITO/MoO_x_-PEDOT:PSS/CH_3_NH_3_PbI_3−x_Cl_x_/PCBM/Bphen/Ag	0.97	21.59	0.754	15.79	[70]
ITO/PEDOT:PSS-Ag/CH_3_NH_3_PbI_3−x_Cl_x_/PCBM/Bphen/Ag	0.93	21.51	0.79	15.75	[72]
ITO/PEDOT:PSS/PolyTPD/CH_3_NH_3_PbI_3_/PCBM/Au	1.05	16.12	0.67	12.04	[30]
ITO/SOHEL/CH_3_NH_3_PbI_3_/PCBM/Al	0.98	16.7	0.71	11.7	[73]
PET/ITO/PEDOT:PSS/PhNa-1T/CH_3_NH_3_PbI_3_/PCBM/Ag	1.03	18.4	0.774	14.7	[74]
ITO/VB-DAAF/CH_3_NH_3_PbI_3_/C_60_/BCP/Al	1.02	18.92	0.78	15.17	[24]
ITO/CPE-K/CH_3_NH_3_PbI_3−x_Cl_x_/PCBM/Al	0.89	20.1	0.77	12.51	[60]
ITO/PT/CH_3_NH_3_PbI_3_/C_60_/BCP/Ag	0.96	22.4	0.78	15.8	[61]
ITO/PBT/CH_3_NH_3_PbI_3_/C_60_/BCP/Ag	1.01	21.1	0.764	16.3	[61]
ITO/PCT/CH_3_NH_3_PbI_3_/C_60_/BCP/Ag	1.01	21.4	0.764	16.5	[61]
ITO/c-OPTD/CH_3_NH_3_PbI_3_/PCBM/C_60_/BCP/Al	1.05	22.4	0.756	17.8	[23]
ITO/PTAA/CH_3_NH_3_PbI_3_/PCBM/C_60_/BCP/Al	1.07	22	0.768	18.1	[23]

**Table 3:** Device characteristics of representative PHJ perovskite solar cells employing graphene oxide and inorganic materials as the supporting layer.

Device Structure	V_oc_ (V)	J_sc_ (mAcm^−2^)	FF	PCE (%)	Ref.
ITO/GO/ CH_3_NH_3_PbI_3−x_Cl_x_/PCBM/ZnO/Al	1	17.46	0.71	12.4	[82]
ITO/GO/PEDOT:PSS/CH_3_NH_3_PbI_3_/PCBM/Al	0.96	17.96	0.76	13.1	[87]
ITO/PEDOT:PSS/GO:NH_3_/CH_3_NH_3_PbI_3−x_Cl_x_/PCBM/Bphen/Ag	1.03	22.06	0.71	16.11	[61]
ITO/BCP/C_60_/CH_3_NH_3_PbI_3_/Spiro-OMeTAD/Au	1.09	23.91	0.73	19.12	[74]
FTO/NiMgLiO/CH_3_NH_3_PbI_3_/PCBM/Ti(Nb)O_X_/Ag	1.072	20.62	0.748	16.2	[83]
ITO/CdS/CH_3_NH_3_PbI_3_/Spiro-OMeTAD/Au	0.977	17.54	0.71	12.2	[90]
ITO/CuS/CH_3_NH_3_PbI_3_/C_60_/BCP/Ag	1.02	22.3	0.71	16.2	[91]
